# Can 2-dimensional echocardiography accurately classify patients with repaired tetralogy of Fallot in possible need of pulmonic valve replacement?

**DOI:** 10.1186/1532-429X-18-S1-P171

**Published:** 2016-01-27

**Authors:** Joyce Woo, Waseem Cossor, Shelby Kutty, Roberto Lang, Amit R Patel

**Affiliations:** 1 University of Chicago, Chicago, IL USA; 2 Advocate Children's Hospital, Chicago, IL USA; 3 University of Nebraska Medical Center, Omaha, NE USA

## Background

Timing of pulmonic valve replacement (PVR) following repaired Tetralogy of Fallot (rTOF) is determined by the severity of right ventricular (RV) dilation. While it is known that cardiovascular magnetic resonance (CMR) can more accurately quantify RV size than 2D-echocardiography (2DE), many centers have not fully integrated serial CMR assessment into the management of these patients, despite guideline recommendations. The aim of this study is to determine the extent to which visual assessment of RV size using 2DE would wrongly classify individuals that should be considered for PVR.

## Methods

CMR and 2DE results were obtained from 61 patients with a history of rTOF. Patients were first classified into events and non-events (i.e. PVR referral or not), based on three cutoffs of RV volume determined by CMR (130, 140 and 150 mL/m2). For each event and non-event group, patients were then reclassified based on presence of greater-than-moderate RV dilation determined qualitatively by 2DE. The net reclassification index (NRI), a measure of prediction improvement, was calculated for each of the three CMR cutoff values. Negative NRI values suggest poorer prediction with 2DE, while positive values suggest improved prediction. Hypothesis testing was conducted with McNemar's method.

## Results

A cutoff of greater-than-moderate RV dilatation determined by 2DE yielded negative NRI for all three CMR event cutoffs (for 130 mL/m2: NRI= -0.998, p = 0.002; for 140 mL/m2: NRI= -1.04, p = 0.004; for 150 mL/m2: NRI= -0.952, p = 0.002). All three NRI were also negative for non-events (Table 1), suggesting 2DE's poorer classification of patients who would not have met RV volume criteria for PVR referral.

**Table 1:** Net Reclassification Index (NRI) for Events and Non-events in Patients with Greater than Moderate Right Ventriular Dilation on 2D-echocardiography

	130 mL/m^2‡^	140 mL/m^2^	150 mL/m^2^
NRI (event^†)^	-0.0476 (p=0.414)	-0.125 (p=0.308)	-0.0769 (p=0.391)
NRI (non-event)	-0.950 (p < 0.001)	-0.911 (p < 0.001)	-0.875 (p < 0.001)
NRI	-0.998 (p=0.002)	-1.04 (p=0.004)	-0.952 (p=0.002)

† events denote patients referred for pulmonic valve replacement, while non-events denote patients where were not referred for pulmonic valve replacement.

‡ indexed right ventricular volume on cardiac magnetic resonance

## Conclusions

Regardless of the RV cutoff value used to trigger referral for PVR, visual assessment of RV size using 2DE results in significant misclassification of both individuals with rTOF who should be referred for PVR and those who should not be referred for PVR. Visual determination of RV size based on 2DE cannot substitute current guidelines recommendations for using CMR to determine timing of PVR. Solutions to increase CMR availability or the identification of appropriate surrogates are needed for the management of these patients.

